# “Awakening consciousness”: Safe Care Linkage Theory From a Mixed Perspective—A Constructivist Grounded Theory Study

**DOI:** 10.1155/jonm/8782807

**Published:** 2026-03-06

**Authors:** Lupei Yan, Xiaorong Wu, Yunman Huang, Li Liu, Fang Wang, Xiuying Hu

**Affiliations:** ^1^ Innovation Center of Nursing Research and Nursing Key Laboratory of Sichuan Province, West China School of Nursing, West China Hospital of Sichuan University, Sichuan University, Chengdu, China, scu.edu.cn; ^2^ Department of Otolaryngology, The First Affiliated Hospital of Chongqing Medical University, Chongqing, China, cqmu.edu.cn; ^3^ School of Nursing, Chengdu Medical College, Chengdu, China, cmc.edu.cn

**Keywords:** constructivist grounded theory, nursing care, older adults, patient safety

## Abstract

**Aim:**

To explore how healthcare professionals, older adults, and caregivers across different contexts conceptualize and implement safe care and develop a substantive theory that reflects their perspectives.

**Background:**

Geriatric care is delivered in a model of ongoing change, with healthcare professionals, older adults, and caregivers highlighting its impact on patient safety. However, there is a shortage of literature on how all care stakeholders conceptualize safe geriatric care.

**Methods:**

Constructivist grounded theory methodology was employed in this study. Between February 2023 and September 2024, semistructured interviews were conducted. Twenty‐five healthcare professionals, 24 inpatients, 19 care workers, and 18 family members from ten hospitals and one primary health center within China participated in the study. Data analysis included initial coding, focused coding, and theoretical coding using constant comparative, memo writing, and field notes.

**Results:**

The substantive theory to emerge from this study was named “Awakening consciousness–Safe care linkage theory.” This theory is informed by four levels: (a) Concept‐motivation level: Generating intrinsic driving force; (b) environment‐institution level: Building a safe care ecosystem; (c) practice‐interaction level: Practicing safe care behavior pattern; and (d) balance‐choice level: Counterbalance the unsafe care crisis. These four levels form the linkage response to implementing safe care for older adults in the hospital, and together with the escort strategy (safety criteria) and progression mechanism (feeling safe) emerging in the data, constitute the linkage ecology of safe care.

**Conclusion:**

The study presents the process of awakening the safety awareness of care stakeholders within geriatric care. Understanding the nature and interrelation of safe care linkage theory could direct practitioners, researchers, and policy‐makers in better efforts to improve the safety of geriatric care. This substantive theory also provides comprehensive theoretical support for future interventions and research to form a safe care ecosystem.

**Implications for Nursing Management:**

The substantive theory provides a theoretical basis for understanding the complex nature of safe care, which illustrates the unsafe care crisis care stakeholders encounter, the criteria they obey, the strategies they use, and the influences of these strategies to practice safe care in complicated situations. Findings will assist nursing leaders in identifying areas to improve care safety and developing and integrating more effective strategies by redesigning optimal management processes to awaken care stakeholders’ safety awareness and take actions suited to the linkage mechanism of a safe care ecosystem.

**Trial Registration:** Chinese Clinical Trial Registry: ChiCTR 2300067421

## 1. Background

In 2000, the Institute of Medicine published a report: “To Err Is Human: Building a Safer Health System,” which brought patient safety into the public eye [1]. Patients deserve safe and high‐quality care, and hospitals are morally and financially invested in achieving it. Despite this duty, safe care for older adults remains a difficult task to consistently complete. In 2023, a study investigating a random sample of 2809 admissions found that preventable unsafe events occurred in 191 of all admissions, and preventable unsafe events with a serious or higher severity level occurred in 29 [2]. Among the preventable unsafe events, 19.7% were serious (prolonged recovery or substantial intervention), 3.3% were life‐threatening, and 0.5% were fatal. This study also found that the percentage of admissions that included at least one unsafe event was higher among older inpatients than younger inpatients. These findings emphasize the importance of geriatric safety and the need for continuing care improvement. Healthcare professionals and caregivers were key to providing safe care and ensuring good care outcomes. The complexity of the environment in which healthcare professionals provide care and the complexity of any given care situation, combined with variable resources, presented a challenge for the researcher wishing to explore how care stakeholders (healthcare professionals, patients, and their caregivers) behave to achieve safe care [3]. However, there is little theory to explain how care stakeholders can conduct care behavior in a comprehensive way to increase the likelihood of safe care across a range of outcomes [4]. Although it is urgent that the patient safety program continues to drive efforts to minimize the risk of unsafe events, there is a real need to develop a new theory instructive to all care stakeholders. Therefore, this study aimed to develop a substantive theory on the nature and process used by care stakeholders to deliver safe geriatric care in the hospital.

A recent study in Harvard Medical Institutions showed that unsafe events occurred in approximately one in four inpatients, and nearly one‐fourth of the events were preventable [2]. In addition, the average length of stay for inpatients with at least one adverse event was more than twice as long as that for inpatients without adverse events (9.3 days vs. 4.2 days), and the average length of stay was 10.8 days for inpatients with at least one preventable unsafe event. Of note, older adults had a higher risk of preventable unsafe events [2]. The complexity of geriatric care, prolonged hospitalization, and human decision‐making in high‐pressure environments contributed to harming care safety [5]. This phenomenon highlights the need for healthcare organizations and care stakeholders to consider their focus and prioritize the importance of safe care. A theory study showed that nurses had a unique responsibility to perform safe care due to a role providing frequent proximity to inpatients, an understanding of risks to all inpatients and comprehension of the care process impacting patients across settings [4]. An extensive body of research supports the significant impact of nurses on care outcomes, including those related to care safety, such as hospital‐acquired infections, falls, pressure ulcers, and other adverse events [6, 7]. The nurse’s perspective on safe care focused more on preventing and handling unsafe care events, emphasizing the implementation outcomes of safe care, while the patient’s perspective differed from it [6, 8].

A constructivist grounded theory showed that patients held perspectives on patient safety that differed from academics and clinicians [8]. While some patients conceptualized patient safety using terms congruent with academic definitions, they mainly described what made them feel safe. Patients’ feelings of safety are derived from multiple care experiences: their friends/family/carers, healthcare professionals, and healthcare organizations. Patients were reassured by the staff’s presence, and they felt safe when they observed healthcare professionals to be motivated and altruistic. Furthermore, patients felt that family members, friends, and care workers contributed to feelings of safety by being advocates and sources of support [8]. While there was a tendency toward ignoring patients’ thoughts on safe care as being too subjective, studies demonstrated that inpatients were willing to give opinions on their care to inform safety improvement [9]. In the past years, calls have been made for patients to play active roles in safe care, and research has shown that patients had positive attitudes about participating in care practice [10]. Arguably, to achieve harmonious partnerships with patients to drive improvements in safe care, it is vital to explore a broader opinion of patient safety that benefits all care stakeholders. Thus, a comprehensive safe care insight should capture the diversity of the care stakeholders’ voices, including the views of seldom heard groups (e.g., caregivers).

Patients and their caregivers are in an important position to support healthcare professionals’ decisions in safe care. A lived experience study showed that patients and their caregivers could provide clinically relevant information to assist healthcare professionals in overcoming challenges associated with making timely and right decisions based on a large and complex information space [11]. A theory study also showed that older adults and caregivers were willing to report safety concerns (e.g., physical discomfort, lack of security, and treatment delays) [3]. Involving patients and caregivers in safe care is one source of creating a resilient system that allows adaptation and flexibility within a complex medical process. This flexibility is needed to improve care outcomes and meet the needs of high‐quality care. However, limited work focuses on patients’ and caregivers’ perspectives of hospital care and the potential influence of patient and caregiver characteristics. This was important when considering that patients and caregivers had a unique insight as they moved within and across system transitions and had the potential to reduce variability experienced from healthcare, which may have an impact on care outcomes [12].

With the aging population anticipated to outpace any other over the next decade and being faced with the challenge of improving safety, there has been an increasing urgency to implement safe care models to achieve age‐friendly health systems. Older adults had more aging‐related high‐risk symptoms, such as frailty, falls, multimorbidity, functional and cognitive impairment, and polypharmacy, which present challenges to implementing previous disease‐based care models [13]. A strong theoretical perspective fosters giving the research greater conceptual depth and breadth while firmly situating it within the discipline. Additional work is needed to support and extend previous research efforts to develop a safe geriatric care theory incorporating broader perspectives. Therefore, this study aimed to explore how healthcare professionals, older inpatients, and caregivers conceptualize and implement safe care and develop a substantive theory to reflect this.

## 2. Methods

### 2.1. Study Design

Constructivist grounded theory methodology, guided by Charmaz [14], was chosen for this study to construct a substantive theory of how older adults, caregivers, and healthcare professionals conceptualize safe care. Grounded theory is based on the principles of symbolic interactionism to explain phenomena, which enables the development of a substantive theory that explains care practice from multiple points of view. By adopting a constructivist stance, the findings acknowledge the researcher’s personal and professional experiences, as well as existing knowledge, in the co‐construction of data with participants [14]. It is through these experiences and interactions that individuals construct and reconstruct their cognition of safe care practice [15]. The study was designed in three stages. Stage 1 included interviews with older adults receiving hospitalization care to understand their care cognition. Stage 2 included field notes of care practice and interviews with older adults’ caregivers to deepen this understanding. Stage 3 included observations of clinical practice and interviews with healthcare professionals with geriatric care experience to develop a substantive theory. The authors adhered to the Consolidated Criteria for Reporting Qualitative Studies (COREQ) in Supporting File 1 [16].

### 2.2. Sample and Setting

Purposive and theoretical sampling was used to recruit participants with care experience and insights to explain the study phenomenon. Findings would have broader relevance if data were collected from multiple clinical settings and spanned various nursing specialties [17]. Therefore, one primary health center and ten Chinese hospitals were used for recruitment.

In Stage 1, we posted a recruitment poster for older adults in the gerontology ward of a Chinese tertiary hospital. Chinese tertiary hospitals generally have almost 1000 beds and are committed to providing high‐quality medical services and undertaking scientific and educational tasks. Inclusion criteria for older adults were (a) aged 65 years and older, (a) being admitted to a hospital for at least 3 days, and (c) being able to speak and understand the study. Participants interested in this study would call us and gain detailed information via face‐to‐face communication. Their caregivers’ information would also be recorded for the next recruitment stage. Purposive sampling of this initial stage chose key informants willing to speak about their experiences, helping maximize variety in patient perspectives. Subsequently, theoretical sampling was used following the first interview, ensuring the emergent concepts and viewpoints from the data, not individuals [14].

In Stage 2, we first contacted the caregivers of participants in Stage 1. The caregiver refers to individuals primarily caring for older adults, including family members and care workers (paid caregivers who have received professional care training). Inclusion criteria for caregivers were (a) aged 18 years and older, (b) taking care of patients for at least 3 days, and (c) being able to speak and understand the study. As the study progressed, the recruitment scope of Stage 2 was not limited to Stage 1. More participants were recruited using a snowball approach to find caregivers with rich care experience to facilitate theoretical sampling principles.

In Stage 3, a recruitment invitation was emailed to healthcare professionals working in the geriatrics ward of Chinese tertiary hospitals as they were considered experts in safe geriatric care. Inclusion criteria for healthcare professionals were (a) registered nurses or practicing physicians, (b) worked in the current ward for ≥ 6 months, (c) had rich experiences in geriatric care, and (d) willing to provide written consent. Participants expressing an interest would receive a research questionnaire to collect individual characteristics and details of their care experiences (e.g., working years and the geriatric subspecialty). These research questionnaires were used as a sample database for theoretical sampling. In keeping with the theoretical sampling principles of grounded theory, further participants were recruited based on emerging codes and ideas in the data analysis [14]. For example, nursing leaders talked about the geriatric care behavior of junior nurses in the interview; then, we chose junior nurses as the next interviewees to expand emerging codes. Moreover, participants were encouraged to recommend suitable colleagues with the potential to enrich the emerging ideas.

**FIGURE 1:**
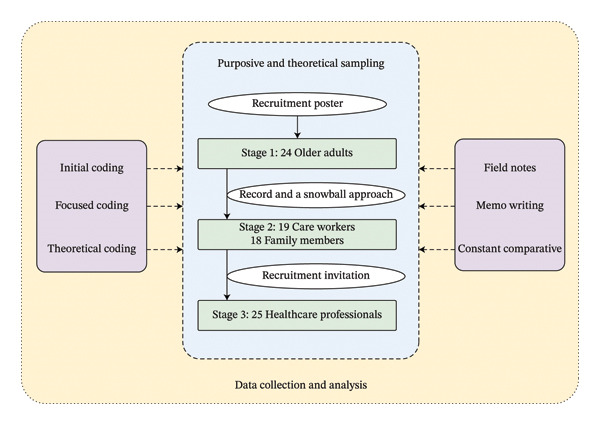
Data collection and analysis process.

Sampling over three stages was successive to enable concurrent involvement in data collection and analysis (Figure 1: data collection and analysis process). Theoretical saturation was reached when no new theoretical insights or categories emerged [18]. After no new theoretical categories emerged, two additional participants were interviewed to confirm data saturation, and data collection ceased.

### 2.3. Data Collection

We conducted face‐to‐face semistructured interviews in three stages between February 2023 and September 2024. Between February and October 2023, we finished the data collection of Stages 1 and 2. We interviewed 61 participants from seven clinical settings (gerontology, orthopedics, internal medicine, neurology, medical rehabilitation, general surgery, and cardiology), including 24 inpatients, 19 care workers, and 18 family members. Before commencing the formal interviews, three pilot interviews were performed to improve the interview guide. Each interview began with “Please describe your hospitalization/care experience related to patient safety,” and emergent lines of inquiry were explored. As with patient and caregiver interviews, theoretical sampling of ideas and categories informed further healthcare professional data collection. Between November 2023 and September 2024, we finished the data collection of Stage 3. We interviewed 25 participants from fifteen clinical settings (e.g., gerontology, neurology, and endocrinology), including 22 nurses, two doctors, and one administrator (medical department). Similarly, two pilot interviews were performed. Each interview started with “What do you think safe care for older adults involves and give examples of how your care practice has contributed to your ideas of this?” and emergent categories were explored through subsequent interview questions and theoretical sampling. Additional topics were added to understand codes and categories from data analysis as the study progressed. Theoretical sampling promoted the recruitment of participants who held “specialist” positions in areas such as geriatric care, chronic disease management, patient safety, and psychological care; this meant that multidisciplinary perspectives were considered. Table 1 (main topic guide) presents the main questions asked during the formal interviews of three stages.

**TABLE 1:** Main topic guide.

• For older adults
◦ Please describe your hospitalization experience.
◦ What measures have you taken to protect your safety?
◦ Are you willing to participate in your care and treatment?
• For caregivers
◦ Please describe your care experience related to patient safety.
◦ How do you understand the safe care of older adults?
◦ What are the factors for the occurrence of unsafe care incidents?
• For healthcare professionals
◦ What do you think safe care for older adults involves?
◦ What are the facilitators and barriers to safe care for older adults?
◦ Give examples of how your care practice has contributed to safe care.
◦ How do older adults and their caregivers participate in patient safety?

All interviews were audiotaped and transcribed verbatim. Participants’ emotions, expressions, and body language related to the study subject were recorded. In addition, we observed the hospital environment and participants’ care behaviors and recorded our feelings and experiences as field notes to provide a deeper understanding and thought for theory construction (Supporting File 2: Examples of field notes). The field notes were also used to depict researcher perspectives and adjust the interview guide as the study progressed. All interviews were performed at a time and location chosen by each participant so as not to interfere with their work or treatment, including their wards, homes, and hospital offices. Although the relationship between some participants and researchers was not established before the study, the study information (e.g., aims and significances, risks and benefits, and confidentiality) was discussed, and written informed consent was obtained before any interview or observation.

We interviewed 86 participants from 11 healthcare institutions. No participants dropped out of the study. Thirty‐six participants were male, and fifty were female. Patients aged 65–91, caregivers aged 26–81, and healthcare professionals aged 25–43. The working years of healthcare professionals ranged from 1 to 25 years (average 8 years), and the care experiences of care workers ranged from 4 to 32 years (average 11 years). Interviews ranged in length from 20 to 90 min (average 40 min). Table 2 presents participant’s demographic characteristics.

**TABLE 2:** Participant characteristics.

Number	Role	Clinical setting	Age	Gender	Years qualified	Interview duration (min)
P1	Older adults	Geriatrics	89	Male	—	42
P2	Older adults	Geriatrics	75	Female	—	35
P3	Older adults	Geriatrics	67	Male	—	30
P4	Older adults	Geriatrics	79	Male	—	30
P5	Older adults	Geriatrics	65	Male	—	40
P6	Older adults	Geriatrics	91	Male	—	39
P7	Older adults	Geriatrics	73	Male	—	20
P8	Older adults	Internal medicine	71	Male	—	20
P9	Older adults	Internal medicine	81	Female	—	31
P10	Older adults	Internal medicine	76	Male	—	27
P11	Older adults	Internal medicine	79	Male	—	39
P12	Older adults	Internal medicine	72	Male	—	21
P13	Older adults	General surgery	77	Female	—	20
P14	Older adults	General surgery	75	Male	—	41
P15	Older adults	Cardiology	76	Male	—	25
P16	Older adults	General surgery	75	Male	—	22
P17	Older adults	Medical rehabilitation	73	Female	—	24
P18	Older adults	Medical rehabilitation	78	Female	—	20
P19	Older adults	Medical rehabilitation	65	Female	—	27
P20	Older adults	Orthopedics	79	Male	—	30
P21	Older adults	Orthopedics	73	Female	—	26
P22	Older adults	Orthopedics	73	Male	—	34
P23	Older adults	Orthopedics	66	Male	—	30
P24	Older adults	Orthopedics	70	Female	—	21
J1	Daughter	Geriatrics	60	Female	—	30
J2	Daughter	Geriatrics	61	Female	—	32
J3	Daughter	Internal medicine	60	Female	—	32
J4	Son	Internal medicine	54	Male	—	40
J5	Son	Internal medicine	56	Male	—	22
J6	Daughter	Internal medicine	58	Female	—	20
J7	Husband	Internal medicine	78	Male	—	31
J8	Daughter	Internal medicine	64	Female	—	30
J9	Husband	Internal medicine	79	Male	—	25
J10	Wife	General surgery	71	Female	—	42
J11	Husband	Orthopedics	73	Male	—	33
J12	Wife	Orthopedics	74	Female	—	33
J13	Son	Orthopedics	45	Male	—	25
J14	Son	Orthopedics	50	Male	—	29
J15	Daughter	Orthopedics	51	Female	—	47
J16	Son	Neurology	49	Male	—	57
J17	Grandson	Internal medicine	26	Male	—	41
J18	Wife	Geriatrics	80	Female	—	50
C1	Care worker	General surgery	60	Female	13	26
C2	Care worker	Geriatrics	53	Male	8	41
C3	Care worker	Internal medicine	51	Male	5	45
C4	Care worker	General surgery	53	Male	10	25
C5	Care worker	Geriatrics	55	Male	18	38
C6	Care worker	Geriatrics	52	Female	4	31
C7	Care worker	Internal medicine	56	Male	10	33
C8	Care worker	Internal medicine	56	Female	8	37
C9	Care worker	Internal medicine	48	Male	4	38
C10	Care worker	Internal medicine	59	Female	32	32
C11	Care worker	General surgery	48	Female	3	90
C12	Care worker	Geriatrics	54	Female	2	39
C13	Care worker	Geriatrics	60	Female	20	87
C14	Care worker	Geriatrics	59	Female	26	42
C15	Care worker	Geriatrics	53	Female	6	69
C16	Care worker	Geriatrics	51	Female	2	85
C17	Care worker	Cardiology	56	Female	10	40
C18	Care worker	Cardiology	52	Male	15	41
C19	Care worker	Cardiology	56	Female	14	35
H1	Head nurse	Geriatrics	36	Female	14	80
H2	Deputy head nurse	Geriatrics	34	Female	10	51
H3	Nurse	Neurology	27	Female	2	53
H4	Nurse	Health management center	30	Male	7	51
H5	Nurse	Endocrinology	26	Female	4	40
H6	Nurse	Neurology	25	Female	1	36
H7	Nurse	Geriatrics	37	Female	12	60
H8	Administrator	Medical department	43	Female	25	34
H9	Head nurse	Neurology	37	Female	13	70
H10	Nurse	Gastrointestinal surgery	29	Female	7	37
H11	Nurse	Ophthalmology	26	Female	1	38
H12	Nurse	Urology	28	Female	5	32
H13	Nurse	Intensive care unit	36	Female	14	86
H14	Nurse	Medical rehabilitation	35	Female	11	43
H15	Nurse	Cardiology	37	Female	15	64
H16	Nurse	Oncology	29	Female	3	36
H17	Nurse	Orthopedic surgery	30	Female	7	37
H18	Doctor	Gastrointestinal surgery	28	Male	2	47
H19	Doctor	Respiratory	33	Male	7	77
H20	Nurse	Orthopedic surgery	29	Female	3	40
H21	Nurse	Geriatrics	26	Male	2	41
H22	Deputy head nurse	Biliary surgery	34	Female	10	40
H23	Nurse	Urology	31	Female	6	65
H24	Deputy head nurse	Gastrointestinal surgery	40	Female	17	49
H25	Nurse	Intensive care unit	30	Female	6	78

### 2.4. Data Analysis

Patients started with P, caregivers started with C, and healthcare professionals started with H. All anonymous transcripts (e.g., H1, P1, and C1) were uploaded into NVivo 20.0 software for data storage and analysis. The first author analyzed the data concurrently using the three phases of coding (initial, focused, and theoretical) in constructivist grounded theory, and the research team reviewed core transcripts to corroborate emerging findings. From the first formal interview, initial coding was performed to fracture the data with line‐by‐line and incident‐by‐incident to find relationships and meanings, outline participants’ insights, and identify behaviors and processes using a constant comparison approach [14]. Key ideas were refined and grouped into common codes to capture descriptions and correlations, and we focused on patterns identified to form concepts and to guide what may be explored during the next interview. These systematic steps centered around identifying and checking ideas and moving back and forth between tentative coding and categorizing to clarify properties within the findings [19]. Focused coding was then used to integrate emerging categories, remaining grounded in the data but including some clustering of ideas. Lastly, theoretical coding was used to outline the logical relationships between categories and concepts. The authors in [14] stated that theoretical coding focused on codes and theorized the data, helping the analysis become more coherent and advance toward an emerging theory. Table 3 presents a coding example of how analysis moved from raw data to concepts with core categories. Furthermore, memos were continuously recorded throughout the data collection and analysis to consider tentative coding, core categories, researchers’ thoughts, and the logic of an emerging theory.

**TABLE 3:** Coding examples.

Initial codes	Focused codes	Categories	Concepts
‐ Blind self‐confidence ‐ Not valuing the importance of care workers ‐ Correcting patients’ misconceptions ‐ Raising safe awareness among patients and their caregivers ‐ Do not pay attention to aspiration ‐ Young people have a higher awareness of safety ‐ Pay attention to the risk factors of patients ‐ Lack of care knowledge among family members ‐ Being familiar with the diagnosis and treatment ‐ Differences in care behavior	‐ Individual cognition ‐ Safety awareness ‐ Care literacy	Role effect	Concept‐motivation level: generating intrinsic driving force
‐ Slowing down the walking speed ‐ Not wearing slippers ‐ Exercise more ‐ Following the doctor’s advice ‐ Following the arrangements of healthcare professionals ‐ Take medicine on time ‐ Care is a learning process ‐ Learning medical knowledge through the internet ‐ Pay attention to the prevention and treatment of thrombosis ‐ Sharing learning resources ‐ Exchanging health experiences ‐ Mutual comfort among patients	‐ Self‐ protection ‐ Compliance ‐ Active learning ‐ Peer education	Subjective initiative	
‐ Assisting in handling discharge procedures ‐ Monitoring changes in the patient’s condition ‐ Reporting abnormal situations ‐ Communicating the condition with healthcare professionals ‐ Checking the patient’s medication ‐ Assisting patients in bathing ‐ Grinding the medicine into powder ‐ Patient admission education ‐ Doctors participate in health education ‐ Explaining the key points of surgery	‐ Patient and their family members participate in patient safety ‐ Caregivers participate in patient safety ‐ Healthcare professionals participate in patient safety	Community of shared responsibility	
‐ Putting the patient’s life first ‐ Patient rehabilitation and discharge ‐ Fall prevention, fall prevention bed, pressure ulcer prevention ‐ The range of patient safety is very wide ‐ The harmonious state of mind, body, and society ‐ Pay attention to psychological safety ‐ Protecting the safety of caregivers ‐ Ensuring patient safety ‐ Implementing various safety measures	‐ Life first ‐ Patient‐centered ‐ Physiological and psychological safety ‐ Safety of caregivers ‐ Goal‐oriented	Holistic care	

### 2.5. Study Rigor

Trustworthiness of the study was enhanced through the criteria of originality, credibility, resonance, and usefulness [14]. Originality was achieved by exploring areas not deeply investigated to gain a broader understanding [14]. Triangulation is a way of improving credibility [14]. This study collected data from a range of sources (patients, caregivers, and healthcare professionals in different clinical settings), and triangulation was achieved through multiple data feedback and team seminars. In addition, we sent transcripts to each participant for checking. Credibility and resonance of interpretations were promoted by feedback from participants about the findings. Finally, usefulness was achieved by providing intervention strategies and practice recommendations for geriatric care.

### 2.6. Ethical Considerations

This study protocol was approved by the Institutional Review Boards of West China Hospital of Sichuan University (No. 20221598). All participants signed informed consent and had anonymous identifiers (e.g., P2 and H13). They could withdraw from the study at any time. Moreover, ethics approval was granted for audio recording, transcribing data, and capturing field notes of the hospital environment and care practice.

## 3. Results

**FIGURE 2:**
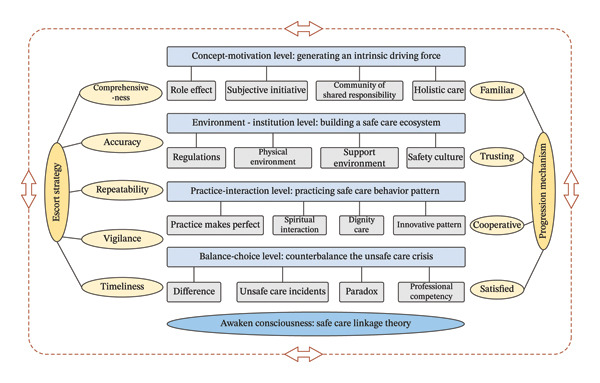
Awakening consciousness: safe care linkage theory.

The substantive theory to emerge from this study was named “Awakening consciousness–Safe care linkage theory” (Figure 2). This theory is informed by four levels: (a) Concept‐motivation level: Generating intrinsic driving force; (b) environment‐institution level: Building a safe care ecosystem; (c) practice‐interaction level: Practicing safe care behavior pattern; and (d) balance‐choice level: Counterbalance the unsafe care crisis. These four levels form the linkage response to implementing safe care for older adults in the hospital, and together with the escort strategy (safety criteria) and progression mechanism (feeling safe) emerging in the data, constitute the linkage ecology of safe care. Each of these concepts, along with the categories that constitute each concept, is described sequentially as follows.

### 3.1. Concept: Motivation Level: Generating an Intrinsic Driving Force

#### 3.1.1. Role Effect

Nurse participants in geriatric departments encountered an everyday concern stemming from older adults’ blind confidence, namely, that patients believed they would not experience unsafe care incidents. This kind of blind confidence phenomenon also existed among caregivers, who thought patients could protect themselves and that they could take good care of older adults.

P14: “I will not have any unsafe care incidents. I am not a dementia patient. I have been hospitalized for a week, and I do not think there is anything unsafe in the hospital.” J11: “I am not worried about unsafe care incidents. I am here to guard her every day, and she is very safe.”

Patients’ perception and evaluation of their abilities could affect their safety awareness, and incorrect individual cognition could lead to unsafe care incidents. Nurse participants believed patients should correctly assess their behavioral abilities and raise safety awareness. At the same time, caregivers should improve their care literacy and take active measures to protect patients.

H6: “Patients feel capable of independently completing many tasks, but they cannot do so. Their wrong perception of themselves has led to security risks.”

C8: “Her family member fed her food, causing her to choke on food, and she could not breathe. I quickly slapped her chest; she vomited out food and started to breathe.”

#### 3.1.2. Subjective Initiative

Subjective initiative refers to the active measures patients take to protect their own safety, which can be summarized into four categories: self‐protection, compliance behavior, active learning, and peer education. Data analysis showed that patients spontaneously engaged in self‐protection behaviors, such as walking slowly, strengthening postoperative exercise, and wearing masks to prevent infectious diseases.

P6: “I walk very slowly, and older adults are afraid of falling.”

J15: “She had some pain in the wound after the surgery, but she still took the initiative to get out of bed and walk slowly.”

Patient participants followed the arrangements of healthcare professionals, regularly took medication, received treatment, and learned disease knowledge through the internet. In addition, patients actively sought advice from experienced patients on symptom management methods and improved their subjective initiative in disease rehabilitation management.

P17: “My doctor gave me medicine for diabetes and hypertension, and I took the medicine according to the regulations. Now, my blood sugar is good.”

P1: “I used to feel dizzy when I woke up, so I learned the “trilogy of waking up” online. Several patients asked me to share this video.”

#### 3.1.3. Community of Shared Responsibility

In safe care practice, patients are the subject, caregivers are the auxiliary subject, and healthcare professionals are the implementing subject. Three groups constitute a community of shared responsibility for safe care practice. The nature of a community of shared responsibility determines the consistency and collaboration of the three groups in executing tasks, namely, the implementation of safe care, which requires the joint participation of patients, caregivers, and healthcare professionals. Many nurse participants encouraged patients to participate in identifying and reporting the treatment process.

H13: “Many preventive measures require the participation of patients and caregivers, and relying solely on nursing staff is not enough.” H16: “When distributing oral medication, I will ask the patient if this is his medication. In fact, patients have a clear understanding of their medication and are unlikely to make mistakes. Involving patients in the process of checking is also a way to protect their safety.”

Data analysis showed that some doctors did not care about unsafe incidents and believed that safe care was the nurses’ job. Nurse participants expressed that patients and their caregivers were more willing to follow doctors’ advice. Doctors’ participation in safe education could increase the safety awareness of patients and caregivers, thereby reducing safety risks.

H22: “It (Patient safety) is often our nurses who do it. Once an unsafe care incident occurs, nurses bear primary responsibility. The effect will be better if doctors can join the safety education and teach patients how to protect themselves.”

#### 3.1.4. Holistic Care

The first level of the emerging theory is the concept‐motivation level, namely, the subjects (patients, caregivers, and healthcare professionals) generate behavioral motivation (intrinsic driving force) driven by concepts to achieve safe care, and the conceptual framework is summarized as holistic care. Holistic care is centered around putting life first and caring for the subjects’ needs. Participants stated that in addition to preventing unsafe care incidents, safe care should include patient’s physical comfort, psychological safety, and social support.

C2: “Patient safety is the most important, and care should be implemented based on patient safety.”

H24: “Patient safety includes both physiological and psychological safety. If you only treat the disease and the patient’s psychological state is poor, they may have difficulty integrating into society after discharge.”

Data analysis showed that safety support should be provided to ensure the safety of caregivers so that caregivers can provide better care.

J12: “I have to guard him here at night, but the hospital only provides a companion bed without a blanket, and I can’t go home to get one. I caught a cold at night. You protect the safety of patients, and you should also protect our safety.”

### 3.2. Environment: Institution Level–Building a Safe Care Ecosystem

#### 3.2.1. Regulations

Data analysis showed that hospitals have established a series of rules and regulations to protect patient safety, such as security regulations, ward management regulations, and check regulations. Nurses informed newly admitted patients about the hospital regulations and helped caregivers understand the department layout. Administrative department personnel also regularly supervised and assessed the implementation level of departmental regulations. However, nurse participants complained that the hospital’s rotation regulations disrupted the normal order of the department, increased their workload, and brought uncertainty to team building.

H15: “We emphasize that newly admitted patients must have a caregiver. Security personnel will check the wristbands of patients and caregivers, and they cannot enter the department without wristbands.”

H2: “The doctors who have just rotated into our department are unfamiliar with the patient. It is nurses who teach them how to issue medical orders, which makes us very tired. After staying for 2 months and becoming familiar with the department, they went to other departments, and a new group of doctors arrived.”

#### 3.2.2. Physical Environment

Field notes showed that large hospitals were filled with age‐friendly designs, including font‐enlarged road signs, guardrails in ward corridors, armrests next to seated toilets, and shower chairs. However, due to the different levels of hospitals and the age of inpatient buildings, there were differences in equipment and facilities among departments. Compared to regular departments, geriatric departments were often located in new buildings with better environments. Participants believed that living in a clean and tidy ward would bring them a pleasant mood, and they hoped that cleaning workers could clean and disinfect the ward every day.

H8: “There are many safety facilities in the toilet, including a one‐click alarm button (SOS), shower chairs, anti‐slip pads, and many safety signs posted on the toilet wall.”

P22: “I was hospitalized in a large hospital before, and the cleaners cleaned and sprayed disinfectant every day. I’ve never seen any cleaners here… Keeping the environment clean is good for patients’ moods.”

#### 3.2.3. Supportive Environment

Data analysis showed that patients were willing to stay in a supportive environment, which can be reflected in friendly communication between healthcare professionals and patients, sincere companionship from caregivers, and mutual help among patients. Due to the busy work of healthcare professionals, caregivers played the main role of accompaniment. When caregivers were fully committed to their work, patients perceived more support, which promoted more interaction in the supportive environment, thus forming a beneficial ecosystem.

C6: “She (the patient) is like my family. I talk to her, walk with her, and make her happy. Our entire ward atmosphere was harmonious, and everyone was happy, like a family.”

#### 3.2.4. Safety Culture

Patients expected to feel a supportive atmosphere in the hospital, and healthcare professionals also needed a good work atmosphere. Some nurse participants stated a staff shortage in their departments, resulting in a tense work atmosphere.

H3: “Every time an unsafe care incident occurs, doctors and nurses blame each other because they are responsible for too many tasks. This tense work atmosphere is a reason for the high staff turnover rate.”

Nurse participants believed that hospitals should expand recruitment efforts, allocate healthcare professionals according to job responsibilities, and establish a fixed specialized team, thus creating a win–win situation and a good safety culture.

H11: “Collaboration between healthcare professionals is a long‐term process. Clarifying each person’s responsibilities gives team members a sense of division of labor, and happy team cooperation can create a good atmosphere.”

### 3.3. Practice: Interaction Level–Practicing Safe Care Behavior Pattern

#### 3.3.1. Practice Makes Perfect

This study found that participants described safe care from a behavioral perspective. Both caregivers and healthcare professionals believed that practicing safe behaviors in care delivery could better protect patients’ safety, which aligns with the principle of “practice makes perfect.” Healthcare professional participants believed that safe care began with health education for patients upon admission. In health education, healthcare professionals understand the patient’s condition, personality traits, and social relationships and then implement precise care. During the shift handover, healthcare professionals also emphasized special shift contents for patients with high safety risks. Caregivers’ understanding of safe care mainly focused on disease observation and companionship.

H19: “First, it is necessary to provide health education to patients and their families. We will give examples of unsafe care incidents for patients who do not cooperate to raise their safety awareness.”

J17: “I have been observing his condition. If there are any abnormalities, I will inform the doctor.”

#### 3.3.2. Spiritual Interaction

Data analysis showed that older patients experienced a heavy emotional burden after hospitalization. They were worried about the illness prognosis and surgery complications and also were concerned that treatment expenses could increase the financial burden on children. The companionship of family members and the care of healthcare professionals can alleviate the psychological pressure on patients. In addition, encouraging patients to express their true feelings can create a spiritual interaction between healthcare professionals and patients, which is more beneficial to their psychological safety.

P20: “I don’t know if I can walk in the future. If I can’t walk, I will need someone to take care of me, which will burden my children.”

P1: “You should pay attention to our mental health, not just treat illnesses. If you talk to me more, I feel better, and my recovery is getting faster.”

#### 3.3.3. Dignity Care

“Dignity care” has emerged in the data analysis, which belongs to psychological support but differs from general psychological care, including patients’ autonomy, preferences, informed consent, and privacy protection. Participants labeled patients with strong autonomy as high‐risk groups for unsafe care incidents, as these patients often overestimated their abilities and believed they could accomplish many things independently.

C4: “I fear caring for older adults who can’t walk but enjoy walking independently.”

H6: “Patients with strong autonomy feel they can act independently and do not like being taken care of, but this group is most high‐risk.”

Healthcare professionals stated that they understood patients’ preferences and respected their autonomy. Before performing each operation, they explained the reasons, sought their consent, and protected their privacy during the operation.

H8: “I met a female patient who felt that her body couldn’t be shown to others. We respected her wishes and explained the reasons for the operation. Finally, she was willing to undergo an operation by a female nurse.”

#### 3.3.4. Innovative Pattern

Data analysis showed that hospitals have taken many measures to help older adults adapt to unfamiliar hospital environments. Caregiver participants often mentioned that the central transportation personnel provided them great convenience.

J12: “It’s much more convenient now. The central transportation personnel informed us of the inspection items and the time the day before. The next day, they took us to the examination on time, and there was no need to wait in line for a long time.”

Nurse participants mentioned that many departments innovated safety care models, such as lost vests for dementia patients, colorful wristbands, and safety grid personnel. Furthermore, hospitals upgraded the internet security control system; set up online disease monitoring, health education, adverse event feedback, and other modules; and designed the green code intelligent notification system.

H19: “Our hospital has a fast track called Green Code. If a patient has a sudden accident, the surrounding security personnel will immediately press one button to call the healthcare professionals, and the nearest department will quickly send staff to rescue the patient. This green code system has saved many patients and won multiple awards in competitions.”

### 3.4. Balance: Choice Level–Counterbalance the Unsafe Care Crisis

#### 3.4.1. Difference

Data analysis showed that differences were an important characteristic of unsafe care incidents. There are differences among older individuals, departments, urban hospitals, and primary health centers. When facing differences, healthcare professionals strive to address unsafe care incidents through existing resources and minimize adverse outcomes.

H18: “The most common issue in our department is catheter dislodgement, and internal medicine department (unsafe care incidents) may be a fall.”

H12: “We previously encountered a patient who had undergone tracheotomy and was transferred from the otolaryngology department. The next day, the patient’s tube fell off, but it was different from the tube in our department, and we didn’t know how to handle it. Later, we asked an otolaryngology nurse to assist with the treatment.”

#### 3.4.2. Unsafe Care Incidents

The fourth level of theory that emerged in this study is the balance‐choice level*:* Counterbalance the unsafe care crisis. Unsafe care incidents, as the focus of hospital quality control, involve multiple contents, such as prevention, response, reporting, cause analysis, consequence handling, and rectification. Data analysis showed that hospitals, healthcare professionals, care workers, and patients have invested a lot of effort in preventing and handling unsafe care incidents to balance the crisis of unsafe care.

H9: “We have invested a lot of effort in preventing unsafe care incidents, such as health education, physical restraint, and age‐friendly design.”

H7: “We initially recorded unsafe care incidents in paper form, but now we report them online through the adverse event system. The system requires detailed information on the reasons, process, handling results, and corrective measures, which are then reported to the nursing department. The nursing department will send staff to supervise whether we have carried out rectification.”

#### 3.4.3. Contradiction

Medical care has a dual nature. Many clinical situations require healthcare professionals to weigh the pros and cons and make the optimal treatment decision for patients, but each decision faces the risk of adverse care outcomes. Because of the particularity of the older patient group, the geriatric medical groups bear greater risks. Healthcare professional participants often felt powerless when faced with dilemmatic medical decisions.

H9: “There are many conflicting issues in clinical practice, such as this cerebral infarction patient who has undergone thrombolysis. When taking medication, you need to consider whether it is to prevent bleeding or thrombosis. It becomes difficult to balance it, and you need to bear the risk of unsafe care incidents.”

Nurse participants also expressed that the complexity of the patient’s condition, the lack of cooperation from family members, and the risks of medication were out of their control. Even though they have taken many preventive measures, unsafe care incidents still occur.

H13: “We have been emphasizing falls and taking many measures to prevent falls, but our department still has fall incidents every year.”

#### 3.4.4. Professional Competency

Nurse participants expressed that older care work brought them heavy work pressure. Under high‐intensity work tasks and pressure, healthcare professionals often enhance their knowledge reserves and clinical ability through professional training. Nurse participants stated that improving their stress resistance and clinical adaptability would increase their professional competency and value. Successfully discharging or rescuing patients would bring them great professional honor.

H16: “Two of my patients are currently at high risk, and I am concerned that their condition may suddenly worsen or that poor care may lead to unsafe care incidents. Nursing is inherently high‐risk, and my nerves are constantly on edge.”

H24: “I used to be a specialist nurse in ostomy, but now I have become a specialist nurse in intravenous therapy. As my specialized nursing skills improved, I could handle more unexpected situations and sometimes felt a sense of professional pride.”

### 3.5. Escort Strategy: Safety Criteria

In the safe care practice, caregivers and healthcare professionals played the role of escorts, ensuring the safety of patients. Safe escorts have many strategies, such as comprehensiveness, accuracy, repeatability, vigilance, and timeliness. This theory summarized these strategies as safety criteria, which were reflected throughout the entire process of safe care. First, healthcare professionals will comprehensively and accurately assess the patient’s illness status, daily activity ability, caregivers’ competency, and social support upon admission.

H15: “When the patient is admitted, we will conduct a comprehensive assessment using various scales such as fall and swallowing function. After the comprehensive assessment, we will educate the patient and their family members to eliminate safety hazards as much as possible.”

H18: “Gastrointestinal surgery requires high accuracy in fluid intake. We need to calculate the amount of fluid replacement based on the patient’s intake and output, but the data recorded by the patients is not very accurate.”

For older adults and high‐risk patients, healthcare professionals increased the frequency of ward rounds and conducted repeated assessments of patients’ various risks. Caregivers also maintained a high vigilance, monitoring and promptly reporting the patient’s abnormal situations.

C13: “Some patients suddenly wheezed, which is a precursor to a cold. I immediately went to tell the doctor.”

These elements are protective strategies for safe care and safety criteria that care stakeholders should keep in mind in geriatric care, running through the entire process of safe care.

### 3.6. Progression Mechanism: Feeling Safe

Caregivers and healthcare professionals implemented safe care under the guidance of safety criteria, and patients, as the main body of safe care, also experienced emotional progression mechanisms, from emotional familiarity to emotional trust to behavioral cooperation and emotional satisfaction, ultimately achieving a sense of safety. The implementation of safe care includes the physiological and psychological safety of patients. Physiological safety can be reflected in the absence of unsafe care events, while psychological safety can be reflected in feeling safe.

H4: “Care is a process of building trust. Patients may be unfamiliar with us initially, but once they become familiar with us, they will trust us and cooperate with our work.” P11: “As soon as I enter the hospital, I feel very safe.”

## 4. Discussion

This study aimed to explore safe geriatric care practices in China and to develop a substantive theory as an explanatory framework. Awakening consciousness is one way to explain how healthcare professionals, older adults, and their caregivers resolve the everyday risks of potential unsafe care incidents. The four linkage levels, escort strategy, and progression mechanism constitute the safe care linkage theory. The theory provides a unique contribution to the literature regarding the generation of the intrinsic driving force to safe care practice, potential influence and risks to care behavior patterns, prevention and management strategies to counterbalance the unsafe care crisis, and the construction of a safe care ecosystem.

The first level of theory is the concept‐motivation level: generating an internal driving force. This indicates that the key to safe care practice is that the care subjects (patients, caregivers, and healthcare professionals) generate an intrinsic driving force for safe care. This study found that patients who participated in safe care experienced a stronger sense of safety. Patients took measures to protect their safety, such as slowing walking, avoiding long‐term bed rest, and actively learning recovery knowledge. These self‐care and self‐protection behaviors reflected the patient’s safety awareness and subjective initiative in safe care [3, 20]. An integrative review showed that patients desired to participate in everyday care and treatment‐related decisions, and being involved in seemingly small decisions made them feel safe and keep control over their own lives [21]. Increasingly, the roles of patients and caregivers have also been advocated as an important component of safe care to prevent unsafe care incidents [12]. Of all medical staff, nurses were in a unique position to enable and encourage the participation of patients and caregivers in their care [22].

In this study, nurses guided patients and caregivers to participate in safe care based on the evaluation results of patients’ self‐care ability and caregivers’ support ability, such as patients participating in medication and treatment identification, caregivers monitoring and reporting patients’ abnormal situations, and family members recording patients’ diet and urine output. However, there is a dearth of literature focusing on how patients and their caregivers participate in safe care with the help of healthcare professionals in China [23]. A United Kingdom team developed and tested the patient safety guide for primary care (PSG‐PC) to support patients and caregivers in identifying the main safety issues where they can make their care safer [12]. Participants expressed great enthusiasm for the PSG‐PC as a tool to support them in being involved in safe care, which showed that it was acceptable and accessible [9]. Patients, caregivers, and healthcare professionals are a community of shared responsibility for safe care. When all care stakeholders’ safety awareness is awakened, they will generate an intrinsic driving force to implement safe care. The third level of this study (the practice‐interaction level) echoes the importance of practicing safe care behavior patterns. Constructing a model for all care stakeholders to participate in safe care and awaken their safety awareness should be the focus of future exploratory studies.

The second level of this theory focuses on the implementation environment and regulations of safe care. From an institutional perspective, Chinese hospitals have generally implemented security and ward management regulations for patients and their caregivers, as well as check and rotation regulations for healthcare professionals. The Chinese rotation regulations originated from the American residency program, aimed at helping newly graduated healthcare professionals transition from theoretical learning to clinical practice [24]. A review concluded that residency programs were beneficial to the new graduate and the hospital’s human resources allocation, but various suggestions for more research were called for regarding residency programs’ influence on teamwork, care practice, curriculum differences, and patient outcomes [25]. Our study found that the healthcare professionals in the Chinese residency programs underwent short‐term rotations in different departments, which led to instability in team building and affected the sustainable development of the safety care ecosystem. A meta‐synthesis also showed that new nurses faced emotional and physical stress during nurse residency programs, which had a negative impact on occupational health and care outcomes [26]. The essence of the residency program was to cultivate healthcare professionals who possessed clinical competency, independently undertook specialized disease treatment, and had good communication, teaching, and research abilities [27]. However, personnel mobility during the programs, the mismatch between departments, the complexity of specialized operations, and the lack of safety awareness among new staff have increased the safety risks [25, 28, 29]. Targeted training of medical staff’s professional abilities and the development of reasonable rotation plans are important for amplifying the advantages of the residency program. Therefore, improving the scientificity and safety of the residency program could be a key point for further studies.

This theory suggests that care stakeholders who possess safety awareness will actively abide by safety regulations, create a safe care ecosystem, and practice safe behavior patterns, namely, the third level of this theory: the practice‐interaction level. With the advent of the digital practice age, medical staff used digital devices to enhance the safety and convenience of geriatric care, and older adults also benefited from it [30]. In this study, older inpatients wore smart devices to monitor vital signs and uploaded discomfort symptoms through the intelligent symptom management system. It is widely recognized that digital technology can hold huge potential to improve the quality of life for older inpatients, and aging in connection with digital technology has grown in awareness [31]. However, involving older inpatients could also be challenging since they were a heterogeneous group with highly diverse characteristics and needs [32]. This study found that older adults were willing to wear smartwatches to monitor vital signs and record exercise data, but they believed that the GPS positioning system exposed their activity traces, and they were unwilling to disclose their disease data. These findings are linked to dignity care at this theory’s third level and are similar to a meta‐synthesis result [10]. Dignity care can be explained as healthcare professionals creating a safe private environment for patients and respecting their preferences [10]. A qualitative study showed that patients felt safe when their dignity was protected and their preferences were respected [33]. Hence, smart devices should be designed to be more customized and user‐friendly to meet older adults’ evolving needs, and medical staff should consider patients’ preferences and protect their privacy when implementing care through intelligent devices.

This theory showed that healthcare professionals needed to weigh complex medical situations and make choices in safe care practice, namely, the fourth level of theory: balance‐choice level (counterbalance the unsafe care crisis). This study found that the unsafe care crisis existed in every aspect of clinical practice due to individual, departmental, and environmental differences. Previous studies also confirmed that older patients had higher risks of unsafe care incidents, and the incidence of unsafe care incidents varied among clinical settings [34, 35]. The rotation regulations aimed to cultivate more comprehensive healthcare professionals and minimize the insecurity crisis caused by differences. However, healthcare has a dual nature, and even experienced medical staff might face paradoxical medical scenarios. Our participants raised a decision dilemma: A cerebral infarction patient who underwent thrombolysis in the acute phase had a high risk of both bleeding and venous thrombosis. It was difficult for medical staff to weigh whether to prevent thrombosis or bleeding first. A mixed study also revealed that physical restraint was a contradiction point between involuntary treatment and safe care, requiring cooperation and understanding from patients and their caregivers [36]. Faced with the complexity and diversity of medical practice, medical staff strive to make choices according to guidelines and professional experience. However, balancing the unsafe care crisis requires the support of all care stakeholders. To our knowledge, there is little research on the contradictions in safe care, and existing studies lack exploration of the causes, decision‐making, and intervention measures for the safe care contradictions. Future research needs to summarize multiple scenarios of safety care contradictions from the perspective of care stakeholders and develop and implement strategies to address safety care dilemmas. This theory will help researchers explore response paths to safety care dilemmas.

The safe care linkage theory demonstrates that the care subject generates intrinsic driving force at the conceptual level, constructs a safe care environment at the institutional level, practices a safe behavior pattern at the practical level, and actively counterbalances the safety care crisis at the balance level. This theory reflects the diversity of subjects and the unity of goals in the safe care practice, supported by previous research [3, 37]. Each operation of safe care requires the care subject to enhance safety awareness and abide by safety criteria. This study summarized comprehensiveness, accuracy, repeatability, vigilance, and timeliness as the safety criteria for safe care practice and the escort strategy for safety care linkage theory. Under the guidance of safety criteria, healthcare professionals, patients, and caregivers achieve a progression mechanism from familiarity to trust to cooperation and satisfaction, ultimately possessing a sense of security. The safe care linkage theory emphasizes that the safe care practice should awaken the safety awareness of care subjects, connect their safety care behaviors, and establish a safety care ecology, ultimately achieving a linkage cycle of safety care. The implementation of care within the care stakeholders is an interconnected process, and the safe care linkage theory provides it with a guiding framework. The four levels of the theory propose different perspectives and emphases on the logic of safe care occurrence. Integrating, designing, and implementing practical models of safe care based on the actual conditions of hospitals and applying them to clinical practice will be both a challenge and an opportunity for future safe management.

## 5. Implications of the Nursing Management

Within nursing policy and research, there is a focus on patient safety, but more emphasis needs to be placed on the safe care theory from a mixed perspective. Understanding the implementation mechanism of safe care for older inpatients would be essential in improving the quality of geriatric care. The substantive theory of “Awakening consciousness” offers a theoretical explanation about the counterbalancing safety risk strategies to practice safely and contribute to care outcomes. Findings will assist organizations in identifying areas to improve care safety and developing and integrating more effective strategies by redesigning optimal management processes to awaken care stakeholders’ safety awareness that strengthens the physiological and psychological safety and quality of patient care. Furthermore, the safe care linkage theory will help medical educators and leaders better understand the link between awareness, environment, practice, and choice from various perspectives and take actions suited to the linkage mechanism of a safe care ecosystem. To our knowledge, no other theory research has been conducted in clinical settings in China to explore safe theory for older adults, and there are many areas for future research as a consequence of this study. Each level (concept‐motivation, environment‐institution, practice‐interaction, and balance‐choice), escort strategy, and progression mechanism, individually or collectively, could be explored further across a range of settings and cultural contexts.

## 6. Limitations

Some limitations of our study should be acknowledged and considered. First, the cross‐sectional nature of the study limited our ability to understand participants’ experiences over time. A longitudinal study might reinforce our understanding of the theory explored in this study. Second, although the study participants represent diversity in care roles, population demographics, and care experience, this is a relatively small sample and does not reflect the entire variety within Chinese hospitals. Lastly, although the substantive theory offers new insight into the views of care stakeholders about safe care, questions remain about whether all concepts of safe care that contribute to the substantive theory have been captured. Experimental and quantitative research should test and improve how the substantive theory can be practically applied and implemented to incorporate safe care into everyday care practice.

## 7. Conclusion

This study presented the theoretical explanation of providing safe care for older inpatients within Chinese hospitals from a mixed perspective. In the safe care linkage theory, the care stakeholders enhance their safety awareness, generate an intrinsic driving force for implementing safe care, practice safe care behavior patterns through compliance with regulations and active interaction, and construct a safe care environment. When facing safe risks, the care stakeholders counterbalance the unsafe care crisis, thus building a virtuous cycle of a safe care ecosystem. Each level of the safe care ecosystem is interconnected to achieve an advanced mechanism for emotions and behaviors under safety criteria. The safe care linkage theory offers a unique insight into how care stakeholders achieve safe care, and the theory also illustrates the unsafe care crisis care stakeholders encounter, the criteria they obey, the strategies they use, and the influences of these strategies on practising safe care in complicated situations. Moreover, this theory presents a novel contribution to the literature, as it aims to avoid oversimplification and does not assume a one‐size‐fits‐all geriatric care model.

Future studies can be conducted around the four levels of the safe care linkage theory, including stimulating the driving forces for care participation among patients and their caregivers, assisting healthcare professionals in mitigating unsafe care crises, increasing the safety awareness of care stakeholders, and establishing a safe care ecosystem. In addition, regarding the clinical challenges identified in this study, research directions such as improving caregivers’ care literacy, optimizing the rotation system for healthcare professionals, and formulating decision‐making guidelines for situations involving medical treatment conflicts are of great clinical significance.

## Funding

This study was supported by the Sichuan Province Science and Technology Support Program (Grant no. 2025ZNSFSC0018).

## Disclosure

See examples of field notes and Consolidated Criteria for Reporting Qualitative Studies checklist in the Supporting Information for comprehensive analysis.

## Conflicts of Interest

The authors declare no conflicts of interest.

## Supporting Information

Additional supporting information can be found online in the Supporting Information section.

## Supporting information


**Supporting Information 1** Supporting File 1: The COREQ.

**Supporting Information 2** Supporting File 2: Examples of field notes.

## Data Availability

Data sharing is not applicable to this article as no datasets were generated or analyzed during the current study.
