# Supplementary material for: “Awakening consciousness”: Safe Care Linkage Theory From a Mixed Perspective—A Constructivist Grounded Theory Study
Source: J Nurs Manag. 2026 Mar 6;2026:8782807. doi: 10.1155/jonm/8782807 (PMC12966611; doi:10.1155/jonm/8782807)
Supplement: Supplementary file 1 — Supporting Information 1 Supporting File 1: The COREQ. [file JONM-2026-8782807-s002.docx]

Consolidated criteria for reporting qualitative studies (COREQ): 32-item checklist

| **No Item** | **Guide questions/description** | **Answer** |
| --- | --- | --- |
| **Domain 1: Research team and reflexivity** |  |  |
| Personal Characteristics |  |  |
| 1. Interviewer/facilitator | Which author/s conducted the interview or focus group? | Yan LP and Wu XR |
| 2. Credentials | What were the researcher’s credentials? E.g. PhD, MD | PhD and MSN |
| 3. Occupation | What was their occupation at the time of the study? | Teachers, researchers, and students |
| 4. Gender | Was the researcher male or female? | Both |
| 5. Experience and training | What experience or training did the researcher have? | Rich experience in grounded theory research |
| Relationship with participants |  |  |
| 6. Relationship established | Was a relationship established prior to study commencement? | Yes |
| 7. Participant knowledge of the interviewer | What did the participants know about the researcher? e.g. personal goals, reasons for doing the research | We interviewed 22 nurses, two doctors, one administrator, 24 inpatients, 19 care workers, and 18 family members. Most of healthcare professionals knew the personal goals, reasons for doing the research.  Although the relationship between some participants and researchers was not established before the study, the study information (e.g., aims and significances, risks and benefits, and confidentiality) was discussed, and written informed consent was obtained before any interview or observation. |
| 8. Interviewer characteristics | What characteristics were reported about the interviewer/facilitator? e.g. Bias, assumptions, reasons and interests in the research topic | Reasons and interests in the research topic of interviewers were reported. |
| **Domain 2: study design** |  |  |
| Theoretical framework |  |  |
| 9. Methodological orientation and Theory | What methodological orientation was stated to underpin the study? e.g. grounded theory, discourse analysis, ethnography, phenomenology, content analysis | constructivist grounded theory |
| Participant selection |  |  |
| 10. Sampling | How were participants selected? e.g. purposive, convenience, consecutive, snowball | Purposive and theoretical sampling |
| 11. Method of approach | How were participants approached? e.g. face-to-face, telephone, mail, email | Face-to-face |
| 12. Sample size | How many participants were in the study? | 86 |
| 13. Non-participation | How many people refused to participate or dropped out? Reasons? | No |
| Setting |  |  |
| 14. Setting of data collection | Where was the data collected? e.g. home, clinic, workplace | Clinic, workplace, or home. |
| 15. Presence of non-participants | Was anyone else present besides the participants and researchers? | No |
| 16. Description of sample | What are the important characteristics of the sample? e.g. demographic data | The sample data sources are diverse, including nurses, doctors, administrator, inpatients, care workers, and family members. |
| Data collection |  |  |
| 17. Interview guide | Were questions, prompts, guides provided by the authors? Was it pilot tested? | Yes |
| 18. Repeat interviews | Were repeat interviews carried out? If yes, how many? | No |
| 19. Audio/visual recording | Did the research use audio or visual recording to collect the data? | Yes |
| 20. Field notes | Were field notes made during and/or after the interview or focus group? | Yes |
| 21. Duration | What was the duration of the interviews or focus group? | Interviews ranged in length from 20 to 90 min (average 40 min). |
| 22. Data saturation | Was data saturation discussed? | Yes |
| 23. Transcripts returned | Were transcripts returned to participants for comment and/or correction? | Yes |
| **Domain 3: analysis and findings** |  |  |
| Data analysis |  |  |
| 24. Number of data coders | How many data coders coded the data | 3088 |
| 25. Description of the coding tree | Did authors provide a description of the coding tree? | Yes |
| 26. Derivation of themes | Were themes identified in advance or derived from the data? | Derived from the data |
| 27. Software | What software, if applicable, was used to manage the data? | Nvivo |
| 28. Participant checking | Did participants provide feedback on the findings? | Yes |
| Reporting |  |  |
| 29. Quotations presented | Were participant quotations presented to illustrate the themes / findings? Was each quotation identified? e.g. participant number | Participant number |
| 30. Data and findings consistent | Was there consistency between the data presented and the findings? | Yes |
| 31. Clarity of major themes | Were major themes clearly presented in the findings? | Yes |
| 32. Clarity of minor themes | Is there a description of diverse cases or discussion of minor themes? | Yes |

-Tong A, Sainsbury P, Craig J. Consolidated criteria for reporting qualitative research (COREQ): a 32-item checklist for interviews and focus groups. Int J Qual Health Care. 2007;19(6):349-357. doi:10.1093/intqhc/mzm042
