# Supplementary material for: “Awakening consciousness”: Safe Care Linkage Theory From a Mixed Perspective—A Constructivist Grounded Theory Study
Source: J Nurs Manag. 2026 Mar 6;2026:8782807. doi: 10.1155/jonm/8782807 (PMC12966611; doi:10.1155/jonm/8782807)
Supplement: Supplementary file 2 — Supporting Information 2 Supporting File 2: Examples of field notes. [file JONM-2026-8782807-s001.docx]

Supplemental 1 Examples of field note

| Number: 1 | Time: June 20^th^, 2024 |
| --- | --- |
| Thoughts：  a. The central transportation staff  b. Shared wheelchairs  c. Auxiliary training equipment | |
| Field site: The lobby of the third inpatient building of a tertiary hospital  Description: The central transportation staff took the patient to do the examination.  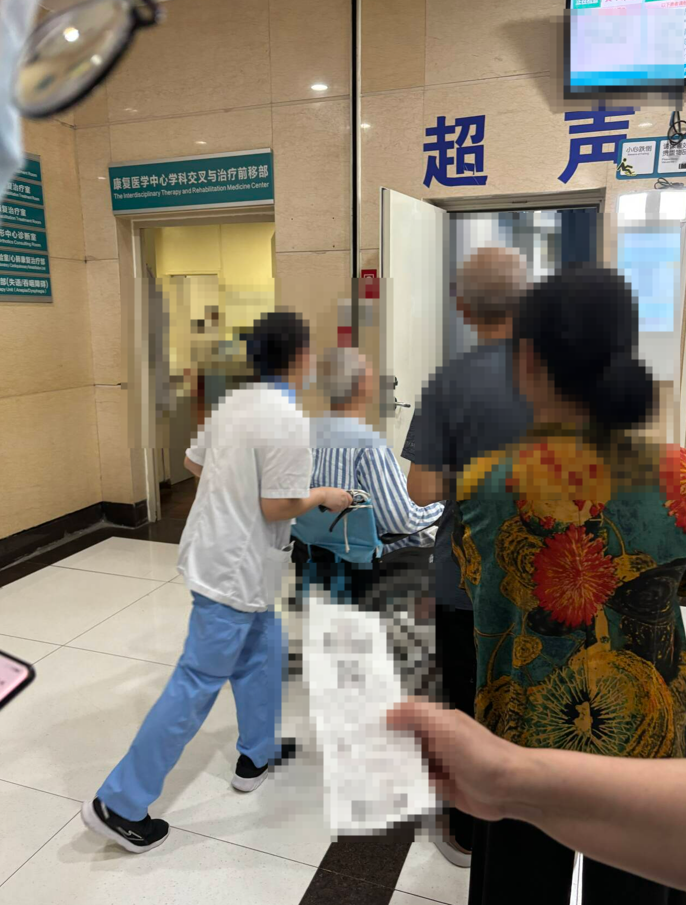 | |
| Field site: The rest area outside the outpatient building of a tertiary hospital  Description: The hospital has set up a self-service wheelchair rental area outside the outpatient building. Patients and their caregivers can scan the QR code to rent wheelchairs according to the operating instructions.  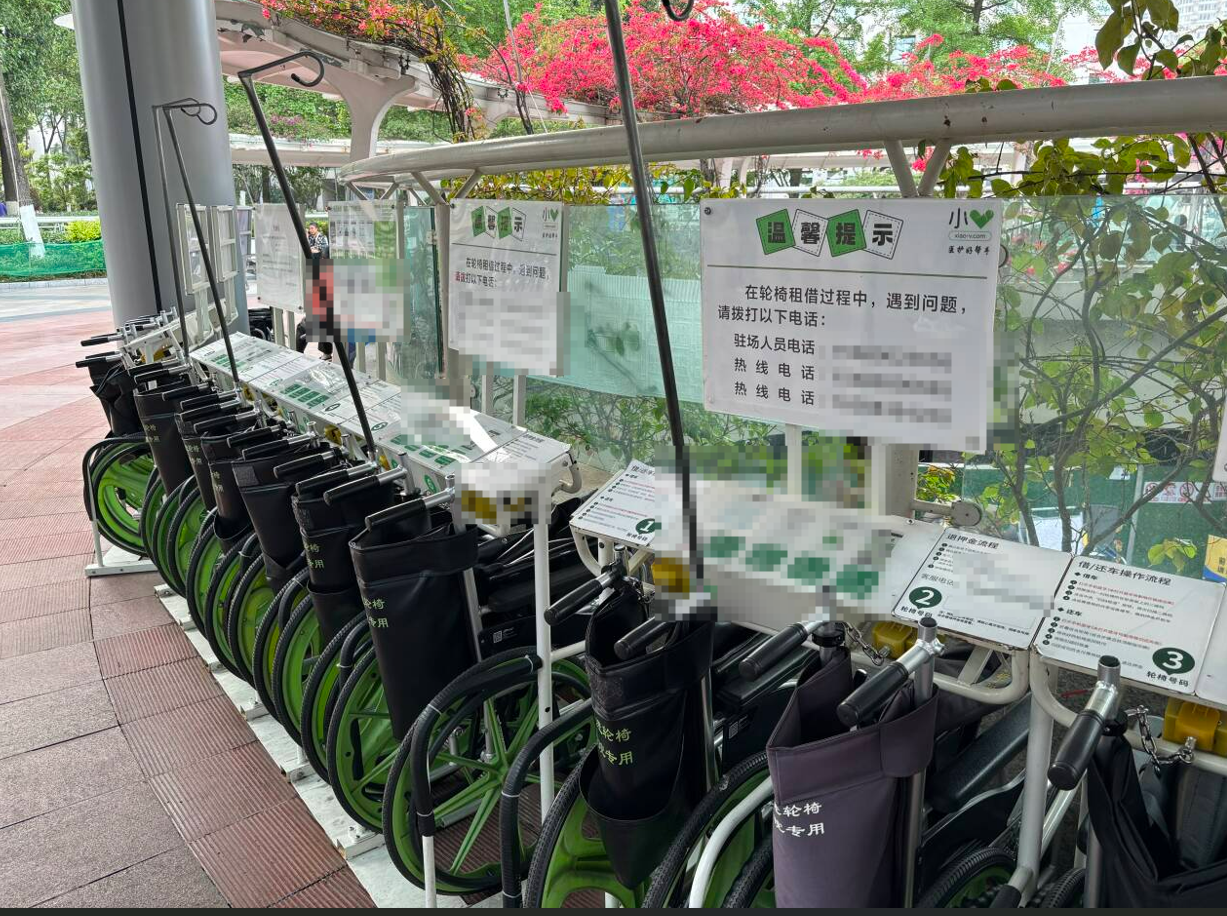 | |
| Field site: The geriatrics department of a tertiary hospital  Description: An older adult unable to walk used the auxiliary equipment for cycling exercise with the help of a care worker.  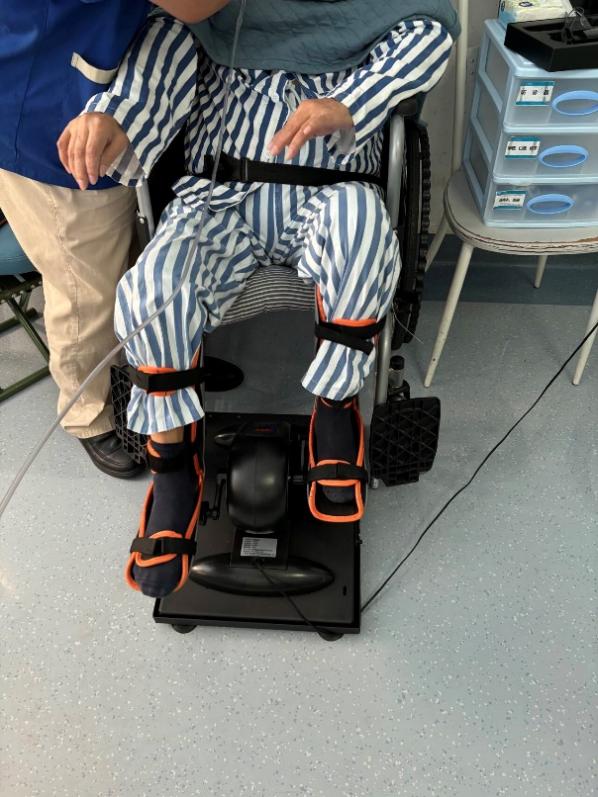  #The photos shooting has obtained the informed consent of the department. | |
